# Purification and Characterization of Alkaline-Thermostable Protease Enzyme from Pitaya (*Hylocereus polyrhizus*) Waste: A Potential Low Cost of the Enzyme

**DOI:** 10.1155/2014/259238

**Published:** 2014-09-18

**Authors:** Mehrnoush Amid, Mohd Yazid ABD Manap, Nor Khanani Zohdi

**Affiliations:** Department of Food Technology, Faculty of Food Science and Technology, Universiti Putra Malaysia (UPM), 43400 Serdang, Selangor, Malaysia

## Abstract

The thermoalkaline protease enzyme from pitaya (*Hylocereus polyrhizus*) waste was purified by a factor of 221.2 with 71.3% recovery using ammonium sulphate precipitation, gel filtration, and cation exchange chromatography. Gel filtration chromatography together with sodium dodecyl sulphate gel electrophoresis (SDS-PAGE) revealed that the enzyme is monomeric with a molecular weight of 26.7 kDa. The apparent *K*_*m*_ and *V*_max_ of the protease were 2.8 mg/mL and 31.20 u/min, respectively. The optimum pH and temperature were 8.0 and 70°C. The enzyme was highly active and stable over a wide pH range (from pH 3.0 to pH 11.0 with the optimum activity at pH 8.0). The protease has broad specificity toward azocasein, casein, hemoglobin, and gelatine. Activity of the enzyme was inhibited by Fe^2+^ and Zn^2+^, while protease activity was increased in the presence of Ca^2+^ and Mg^2+^ and Cu^2+^ by factors of 125%, 110%, and 105%, respectively. The alkaline protease showed extreme stability toward surfactants and oxidizing agent. The purified protease exhibited extreme stability in the presence of organic solvents and inhibitors. In addition, the enzyme was relativity stable toward organic solvents and chelating agents, such as ethylenediaminetetraacetic acid (EDTA). The enzyme, derived from pitaya peel, possesses unique characteristics and could be used in various industrial and biotechnological applications.

## 1. Introduction

Proteolytic enzymes (EC 3.4) are a group of enzymes, the catalytic function of which is to hydrolyze the peptide bonds of proteins. Proteases are commercially important enzymes, and it has been reported that approximately 60% of the total worldwide market of enzymes is comprised of proteases [1]. Proteases of plant origin perform many vital functions, ranging from the mobilisation of storage proteins during germination to the initiation of cell death and senescence [2]. Plant-derived proteases have been used in various industries, such as food, detergent, pharmaceutical, leather, and biotechnological application due to their high stability in extreme conditions, substrate specificity, good solubility, and activity over wide temperature and pH ranges. Plant proteases, being one of the largest groups of proteolytic enzymes, are involved in numerous regulatory processes in plants. However, despite being the largest class of proteases, the functions and regulatory roles of plant proteases are poorly understood, most likely due to a lack of identification of their physiological substrates [3].

Apparently, most of the isolated and characterized plant proteases have been classified as cysteine proteases, which are widely used in several processes in the food industry. The major drawback in the use of cysteine proteases is that thiol proteases are reduced by metal ions and air oxidation; thus, there is a need for chelating agents and a milder reducing environment for activation, therefore making the use of cysteine proteases cost inefficient [4]. Hence, the proteases are more economical for industrial use [5]. Therefore, the search continues for new plant proteases, the physiological roles of which we hope to understand with the purpose of discovering solutions that are industrially applicable and cost effective. Pitaya plant peel could be a potential source of proteases due to easy purification methods associated with it, low levels of interfering substances during purification, and good yield of proteases [4]. The red pitaya fruit (*Hylocereus polyrhizus*) has recently drawn the close attention of growers worldwide because of its economic value and potential health benefits [6]. The peel of the red pitaya fruit comprises approximately 33% of the entire fruit, but the peel is not currently used in any commercial application. The peel contains a valuable natural protease that can be used as a rich, natural, and abundant media source for commercial production of the enzyme. Although red pitaya is a rich, natural, and cost effective source of protease, no study has the characterization to the knowledge of this researcher. The present study thus investigates the purification and characterization of protease enzyme from red pitaya peel, including the kinetic and catalytic properties of the purified enzyme.

## 2. Material and Methods

### 2.1. Plant Material and Chemicals

Red pitaya fruits (*Hylocereus polyrhizus*) were purchased from Pasar Borong (Selangor, Malaysia). Ripened pitaya fruits were selected based on the size uniformity at the same stage of ripening free of visual defects. The fruits were stored in a cold room at 4°C until use for the extraction procedure. All chemicals and reagent were in analytical or electrophoresis grade. SP-Sepharose, Sephacryl S-200, Bradford Reagent, BSA, DTNB, PMSF, EDTA, ovomucoid, iodoacetic acid, bestatin, *β*-mercaptoethanol, PMSF, and trichloroacetic acid (TCA) were obtained from Sigma Chemical Co. (St. Louis, MO, USA). Tris-HCL, Triton X-100, Tween-80, SDS, casein, haemoglobin, acetone, ethanol, isopropanol, and methanol were obtained from Merck (Darmstadt, Germany).

### 2.2. Extraction of Thermoalkaline Protease

Fresh pitaya fruits (2 Kg) were cleaned and rinsed thoroughly with sterile distilled water and dried with tissue paper. The peels of pitaya were removed and chopped into small pieces (1 cm^2^ each, 1 mm thickness); then, they were quickly blended for 2 min (Model 32BL80, Dynamic Corporation of America, New Hartford, CT, USA) with sodium acetate buffer at pH 5.0 with ratio 4 : 1, at temperature 2.5°C. The peel-buffer homogenate was filtered through cheesecloth and then the filtrate was centrifuged at 6000 rpm for 5 min at 4°C and the supernatant was collected [7]. Supernatant (crude enzyme) was kept at 4°C to be used for the purification step.

### 2.3. Purification of Thermoalkaline Protease

A combination of ammonium precipitation, desalting, SP-Sepharose cation exchange chromatography, and Sephacryl S-200 gel filtration chromatography was employed to separate and purify the protease enzyme from the pitaya peel. The crude enzyme was first brought to 20% saturation with gradual addition of powdered ammonium sulphate and allowed to stir gently for 1 hr. The precipitate was removed by centrifugation at 10,000 rpm for 30 min and dissolved in 100 mM Tris-HCL buffer (pH 8.0). The supernatant was saturated with 40%, 60%, and 80% ammonium sulphate. The precipitate of each step was dissolved in a small volume of 100 mM Tris-HCL buffer (pH 8.0) and dialyzed against the 100 mM Tris-HCL buffer (pH 5.0) overnight with frequent (6–8 interval) buffer changes and centrifuged again. The dialyzed suspension after ammonium sulfate precipitation was subjected to cation exchange chromatography on SP-Sepharose fast flow column preequilibrated with 100 mM Tris-HCL buffer at pH 8.0.

The column was washed with the same buffer until no protein was detected in the eluate. The bound proteins were eluted with Tris-HCL buffer at pH 8.0 using a linear gradient of NaCl from 0 to 0.9 M. The flow rate of 1 mL/min was maintained, and 5 fractions of 1.0 mL each were collected. All of the fractions were examined for proteolytic activity, protein content, and homogeneity using enzyme assay, absorbance at 280 nm, and SDS-PAGE, respectively. The active and homogenous fractions from the cation exchange were pooled and submitted to one cycle of gel filtration on a Sephacryl S-200 column preequilibrated with 25 mM Tris-HCL at pH 8.0 containing 0.6 M NaCl. The column was eluted by 100 mM Tris-HCL buffer (pH 8.0) to wash the unbound proteins. The bound proteins were eluted with linear salt gradients of 1%, 2%, 3%, 4%, and 5% NaCl in the same buffer. All of the fractions were analyzed as described above. The active and homogenous fractions were pooled, concentrated, and stored at 4°C for further analysis.

### 2.4. Proteolytic Activity Assay

The proteolytic activity of purified protease was measured according to the method described by Zanphorlin et al. [8] with some modification. The reaction mixture contained 1 mL of 0.5% (wv^−1^) azocasein prepared in 100 mM Tris-HCl (pH 8.0) buffer and 0.1 mL of enzyme. The mixture was incubated in a water bath at 80°C for 1 h, and 10% (wv^−1^) of 0.3 mL of trichloroacetic acid (TCA) was added to stop the reaction, followed by centrifugation at 10,000 rpm for 10 min at room temperature (Microfuge 18 centrifuge, Beckman Coulter, Inc., Krefeld, Germany). The absorbance of the TCA-soluble supernatant was determined at 410 nm using a spectrophotometer (BioMate-3, Thermo Scientific, Alpha Numerix, Woodfield Dr, Webster, NY, USA). One unit of proteolytic activity is defined as the amount of enzyme causing an increase in absorbance of 0.01. The specific protease activity was expressed as enzyme activity (U) per mg of protein. The control was run by substituting the enzyme with the same volume of enzyme extract heated in a boiling water bath for 30 min for inactivation of the enzyme.

### 2.5. Determination of Protein Concentration

Protein concentration was determined by the Bradford [9] method and BSA was used as standard.

### 2.6. Determination of Purity and Molecular Weight of Purified Protease

SDS-PAGE was performed on a minivertical gel electrophoresis unit (Amersham Biosciences) using 15% acrylamide separating gel in the presence of 0.1% SDS and 4% acrylamide stacking gel containing 0.1% SDS according to the method described by Laemmli [10]. The SDS reducing sample buffer and tank buffer were 0.5 M Tris-HCl (pH 6.8) containing 2% SDS and Tris-glycine (0.025 M Tris-HCl, pH 8.3; 0.192 M glycine) in the presence of 0.1% SDS, respectively. Electrophoresis was performed at room temperature, and the run was conducted at 15 mA and 250 V for the stacking gel and 30 mA and 250 V for the resolving gel. Proteins in the enzyme solution were denatured by heating the sample (3.47 ng of protein (16 *μ*L)) with 4 *μ*L of SDS reducing sample buffer at 100°C for 5 min before loading 15 *μ*L into the gel. After electrophoresis, protein bands on the gel sheets were visualized by silver staining using the procedure described by Mortz et al. [11].

### 2.7. Optimum Temperature and Temperature Stability of the Protease Enzyme

The effect of temperature on protease activity was determined by incubation of the reaction mixture (azocasein and purified enzyme) at temperature ranging from 20 to 100°C (at 10°C intervals). Determination of protease activity was performed using the standard assay condition as described above. Temperature stability of the protease was investigated by incubating the enzyme in 50 mM Tris-HCL (pH 8.0) within temperature range of 10 to 100°C for 1 h. The residual enzyme activity was determined by azocasein at pH 9.0 and 70°C for 1 h [12].

### 2.8. Optimum pH and pH Stability of the Protease Enzyme

The optimum pH of the protease was determined by measuring the azocasein hydrolyzing activity ranging from 3.0 to 12.0 at the optimum temperature. The residual enzyme activity was determined under standard assay condition. The appropriate pH was obtained using the following buffer solutions: 100 mM sodium acetate buffer (pH 3.0–5.0), 100 mM phosphate buffer (pH 6.0-7.0), 100 mM Tris-HCl buffer pH (7.0–9.0), and 100 mM carbonate (pH 10.0-11.0). The pH stability of the purified protease was determined by preincubating the enzyme at different pH for 1 h at 70°C. Then, the residual protease activity was determined under optimum conditions of pH and temperature as described earlier. The activity of the enzyme before incubation was regarded as 100% activity. The results were expressed in averages (duplicates) with an estimated error of ±10% [13].

### 2.9. Effect of Metal Ions on the Protease Activity

The effect of various metal ions on the protease activity was determined in the presence of 10 mM of Li^+^, K^+^, Na^+^, Sn^2+^, Zn^2+^, Fe^2+^, Mg^2+^, and Ca^2+^. The initial concentration of the metal ions was prepared by dissolving them in deionised water. Purified enzyme (100 *μ*L) was preincubated with 100 *μ*L of 10 mM of the metal ion at the optimum temperature and pH for 1 h in a water bath. Then, the enzyme-metal ions mixtures were incubated with 1 mL of 0.5% (wv^−1^) of azocasein as the substrate in Tris-HCl buffer (pH 8.0) for 20 min in a water bath at 70°C. Residual activity was determined after terminating the reaction with 0.3 mL of 10% (wv^−1^) TCA, as described in the standard protease assay earlier.

### 2.10. Effect of Inhibitors, Organic Solvent, and Surfactant and Oxidizing Agents on the Protease Activity

The impact of enzyme inhibitors on the enzyme activity was studied using 5 mM PMSF, ovomucoid, iodoacetic acid, bestatin, DTNB, EDTA, and *β*-mercaptoethanol. The effect of some organic solvents such as acetone, ethanol, isopropanol, and methanol on protease activity was also investigated. In addition, the effects of chemicals on the enzyme activity were studied using 2 M H_2_O_2_ as oxidizing agent as well as 5% Triton X-100, 5% Tween-80, and 10% SDS as ionic and nonionic surfactant agents on the protease activity determined [8, 14]. The enzyme was incubated with each reagent for 30 min at 70°C in water bath and then residual activity of the enzyme was determined as described earlier and expressed as a percentage of the activity obtained in the absence of the reagents.

### 2.11. Substrate Specificity

The substrate specificity of the purified enzyme was determined using various natural substrates, namely, casein, hemoglobin, BSA, and gelatine, according to the method described by Khan et al. [15]. The above substrates were prepared individually by dissolving 0.5% (w/v) in 100 mM Tris-HCl buffer (pH 8.0). The activity obtained with azocasein was used as the control (100%). According to Khan et al. [15], the absorbance of the TCA-soluble supernatant was found to be 410 nm for azocasein and 280 nm for casein, haemoglobin, BSA, and gelatine using a spectrophotometer (BioMate-3, Thermo Scientific, Alpha Numerix, Woodfield Dr, Webster, NY, USA).

### 2.12. Determination of *K*_*m*_ and *V*_max⁡_

Different concentrations of azocasein (5–50 *μ*L) in Tris-HCl (30 mM, pH 8.0) were incubated with the enzyme for 10 min at 70°C. The enzyme concentration was kept constant (20 *μ*g protein mL^−1^ extract) and protease activity assay was performed at optimum reaction conditions. Initial velocities (*V*_0_) were determined at all substrate concentrations and the *K*_*m*_ and *V*_max⁡_ values were calculated from the double reciprocal plot [16].

### 2.13. Experimental Design and Analysis

All the experiments were organized using a completely randomized design with three replicates, repeated twice for reproducibility. The analysis of the experimental data with two-way analysis of variance (ANOVA) was conducted followed by the Fisher multiple comparison test at *p* < 0.05. The least significant difference (LSD) test was used to determine if there were significant differences among the samples.

## 3. Result and Discussion

### 3.1. Purification of the Protease from Red Pitaya

**Figure 1:**
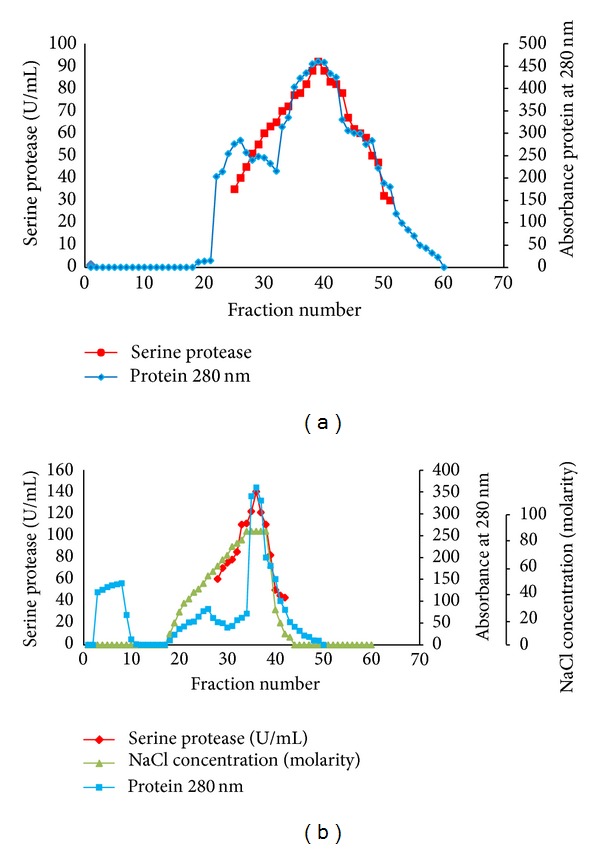
Cation exchange and gel filtration chromatography plots. (a) shows the cation exchange chromatography on SP-Sepharose (when the column was equilibrated with Tris-HCL at pH 8.0). The protein of interest eluted in the unbound samples. (b) The nonretained fraction from SP-Sepharose 200 was loaded to gel filtration chromatography on Sephacryl S-200. Column was eluted with linear salt gradient in the same buffer.

**Figure 2:**
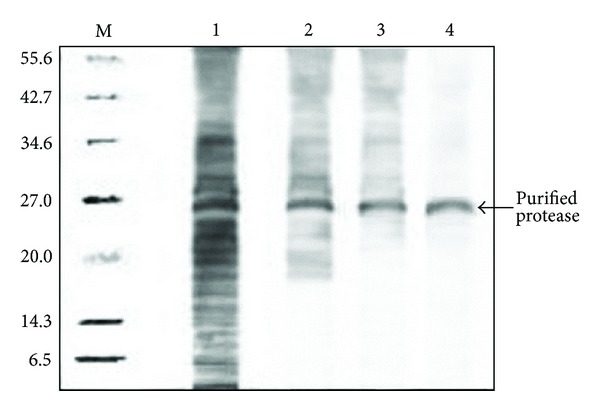
SDS-PAGE of the purified protease. M: standard protein markers; lane 1: crude enzyme; lane 2: ammonium sulphate-precipitated enzyme; lane 3: purified enzyme on SP-Sepharose (cation exchange); lane 4: purified enzyme on Sephacryl S-200 (gel filtration).

A single protein with the protease activity was purified from the red pitaya peel by ammonium sulphate precipitation, cation exchange chromatography on a SP-Sepharose column, and gel filtration chromatography on Sephacryl S-200.  Table 1 summarizes the study of purification of the protease from pitaya peel. The extracted enzyme was precipitated with ammonium sulphate and, based on the results, 60–80% saturation produced the highest purification by a factor of 9.4 with a yield of 83.2% among the other ammonium sulphate concentrations. The concentrated fraction was then loaded onto the cation exchange chromatography column (SP-Sepharose). The enzyme was eluted from the column with a salt concentration of 1.5 M NaCl. The enzyme activity and proteins were found in one peak after elution (Figure 1(a)). The protease from red pitaya peel was purified by a factor of more than 104.2 with a 74.1% yield, with its specific activity equal to 1312.9 U/mg proteins (Table 1). The active fractions of cation exchange chromatography were separated by Sephacryl S-200 gel filtration chromatography (Figure 1(b)). After this step, protease was purified by a factor of 221.2 with a recovery of 71.3% and a specific activity of 2787.1 U/mg proteins, respectively (Table 1). The gel filtration chromatography technique and ion exchange chromatography used in this study have also been used successfully for the protease purified from latex* of Euphorbia milii* from sweet potato roots [17, 18]. It can be observed that the enzymatic activity was eluted in one peak, which coincided with the peak of protein. Fractions of this peak (35–42) were collected and concentrated. The purified protease was homogenous as it gave a single protein bond on SDS-PAGE. The molecular weight of the protease by SDS-PAGE was approximately 26.7 kDa (Figure 2). The molecular weight obtained by Sephadex G-200 and DEAE-Sephadex column chromatography was also approximately 26.7 kDa (Figure 2). It can be observed that the enzymatic activity was eluted in one peak, which coincided with the peak of protein. Fractions of this peak (46–49) were collected and concentrated. The purified protease was homogenous as it gave a single protein band on SDS-PAGE. Molecular weight of the protease by SDS-PAGE was approximately 26.7 kDa (Figure 2). The molecular weight obtained by SP-Sepharose and Sephacryl S-200 column chromatography was also approximately 26.7 kDa (Figure 2).

### 3.2. Optimum Temperature and Thermal Stability of the Purified Protease

**Figure 3:**
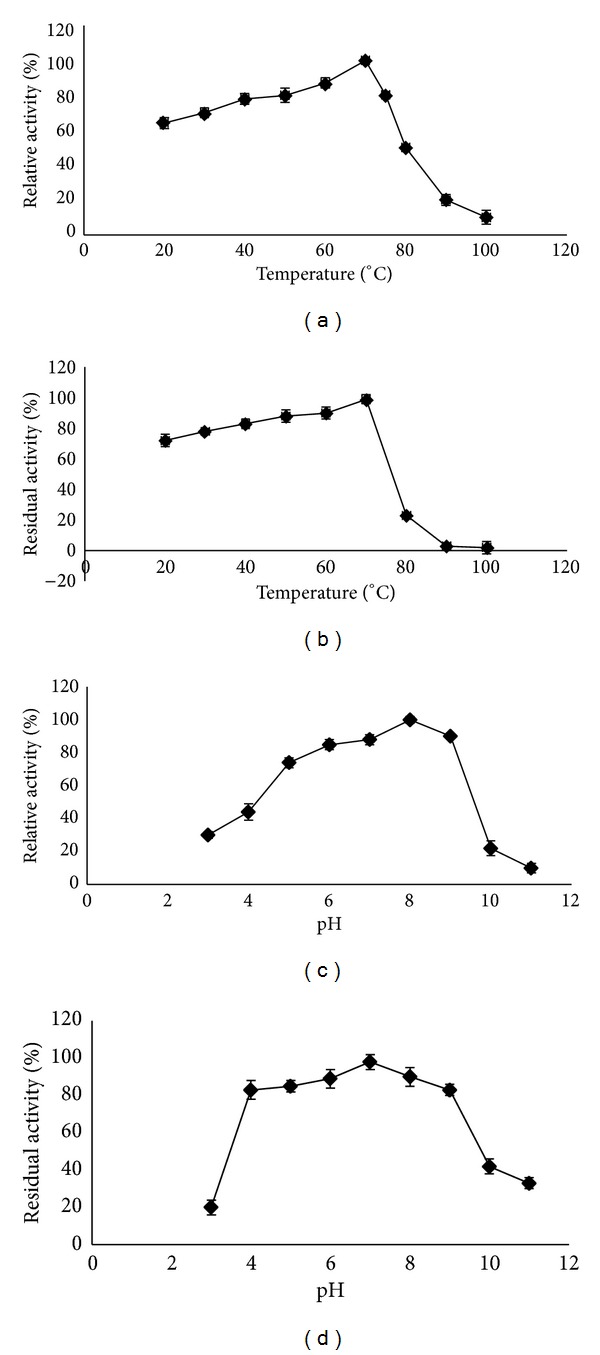
The optimum temperature (a), thermal stability (b), optimum pH (c), and pH stability (d) of purified thermoalkaline protease were investigated.

The purified protease from red pitaya peel was active and stable throughout a wide temperature range (20°C to 75°C). The temperature for the maximum protease activity was 70°C. At both 80 and 90°C, the protease was quite active, with almost 60% and 35% activity, respectively. Therefore, the results reveal that the optimum temperature for the enzyme is 70°C (Figure 3(a)). Analysis of the thermal stability of the protease showed that the enzyme retained more than 90% of its activity in the range of 20 to 80°C, but the enzyme activity was significantly (*p* < 0.05) decreased at temperature above 80°C. The residual activity of the purified enzyme at 80°C was 23%, but above that temperature no detectable enzyme activity could be determined (Figure 3(b)). This phenomenon could be due to the denaturation of the enzyme at a heightened temperature. There are some reports in agreement with this study for isolated protease from some plant sources [19]. Therefore, the purified protease from pitaya peel showed the high thermostability. It should be mentioned that thermostability of the enzyme is one of the good characteristics of the protease. In addition, thermostable enzyme can decrease the risk of contaminants at high temperature in industries and also cost of external cooling and the increased substrate solubility, allowing for higher concentrations of low solubility materials and a lower viscosity of liquids and it may also be useful in mixing.

### 3.3. Effect of pH on Activity and Stability of the Purified Protease

In the pH activity experiments, the protease was observed to be approximately 75% active in the pH range of 7.0 to 9.0 with 100% activity at pH 8.0. At pH levels of 3.0 and 10.0, the protease activity was reduced to 30% and 22%, respectively. The protease was thus stable (30–100% of maximum activity) throughout the entire pH range that was studied. The enzyme exhibited the highest stability (85%) in the pH range 4.0 to 10, with 100% stability at pH 8.0 (Figure 3(c)). The residual activity sharply decreased at pH levels above 10.0, with 33% of the initial activity of the enzyme observable at a pH of 11.0 (Figure 3(d)). The remarkable activity and stability over a wide pH range reveal the highly alkaline nature of this protease, which makes it suitable for applications in alkaline environments and with detergents. It should be noted that the purified protease exhibited good stability in the wide range of pH from acidic to alkaline, while, the activity of the purified enzyme was higher in alkaline pH. These results agree with the protease activity from* Euphorbia milii* where the maximum activity was recorded at pH 8.0, and the residual enzyme activity markedly decreased at pH levels above 10.0 [20].

### 3.4. Effect of Metal Ions on the Purified Protease

The influence of various metal ions on the purified enzyme is presented in Table 2. The activity of the protease was not significantly (*p* > 0.05) affected by 10 mM of Li^+^, Na^+^, K^+^_,_ and Sn^2+^, while the activity of enzyme was decreased in the presence of Zn^2+^ and Fe^2+^. Maximum inhabitation of approximately 38% and 52% was observed with 10 mM Zn^2+^ and Fe^2+^. The enzyme activity was significantly enhanced in the presence of Mg^2+^, Ca^2+^, and Cu^2+^ up to 110%, 125%, and 105%, respectively. Based on the results, although Ca^2+^ ions stabilized the enzyme at high assay temperature and increased enzyme activity and stability, they were not required for the activity of the protease from red pitaya peel. The lack of a need for Ca^2+^ ions for protease activity is one of the desirable characteristics of the enzyme. Because the enzyme has these characteristics, it is suitable for the use in various types of industries especially in food processing, beverage production and clarification, sewage treatment, and many other applications [21]. Tripathi et al. [22] reported that the inactivation of the enzyme by these metal ions may be due to their binding to the catalytic residues in the active site of the enzyme.

### 3.5. Effect of Inhibitors, Organic Solvent, and Surfactant and Oxidizing Agents on the Purified Protease

Based on the results shown, in Table 2, the inhibitor of trypsin like ovomucoid had no effect on the protease activity as well as inhibitors against cysteine protease. Similarly, the use of reducing agent *β*-mercaptoethanol did not have any significant (*p* > 0.05) effect on its activity, and we thereby infer that the protease was not a cysteine or trypsin type. However, there was strong inhibition of the enzyme in the presence of the protease inhibitor phenylmethanesulfonyl fluoride (PMSF). Meanwhile, thiol reagent (i.e., 5,5′-dithiobis-2-nitrobenzoic acid, DTNB) only partially influenced the activity of the purified enzyme. In addition, the activity of the enzyme increased by 15% in the presence of 10 mM of ethylenediaminetetraacetic acid (EDTA). This phenomenon could be due to the structural rigidity of the thermostable protease and the tight bounding of Ca^2+^ and other divalent cations to the enzyme, as the removal of divalent cations from enzymes by EDTA treatment is notably difficult.

There have been similar results demonstrating the difficulty of removing Ca^2+^ and Zn^2+^ from extracellular *α*-amylase from* Pyrococcus furiosus* using EDTA treatment at temperature below 70°C due to the tight binding of the cations to the enzymes [23, 24]. There was the presumption that increased conformation flexibility, due to the effect of chelators on the enzyme's native conformation, was responsible for the increase in enzyme activity. EDTA is commonly present in detergents because it has been found to be a very suitable additive due to the effect of EDTA on proteins. It was found that the protease activity was not affected significantly (*p* > 0.05) by other inhibitors and hydrophilic solvents such as acetone, ethanol, ethyl acetate, and isopropanol (Table 2). The enzyme was also highly stable in the presence of the nonionic surfactants such as Triton X-100 and Tween-80 (Table 2). The enzyme also showed great stability in the presence of strong ionic surfactant (SDS), retaining 73% of the initial activity when the enzyme was incubated in the presence of 5% SDS in the reaction mixture. The enzyme also showed good stability in the presence of an oxidizing agent (H_2_O_2_) and retained 62% of the initial activity after being incubated with 2 M (v/v) of hydrogen peroxide for 1 h (Table 2). The fact that the enzyme was not sensitive to the reagents could indicate that the protein may have a well-packed structure and that its native confirmation is rigid [25].

### 3.6. Substrate Activity

**Figure 4:**
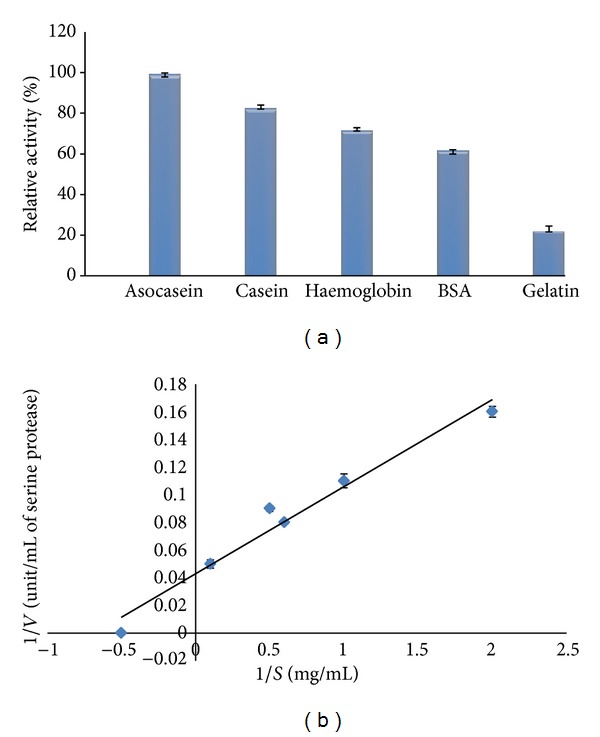
Substrate specificity (a) and kinetic properties (b) of the protease were investigated.

The substrate specificity of the purified enzyme showed that it was active on a variety of modified substrates: azocasein and natural proteins such as casein, haemoglobin, bovine serum albumin (BSA), and gelatine, as shown in (Figure 4(a)). The protease exhibited the highest activity towards azocasein. The enzyme also showed higher proteolytic activity on casein and haemoglobin, at 83% and 72%, respectively, compared with activity on azocasein as the substrate. This enzyme partially hydrolysed BSA and gelatine, with gelatine serving as the poorest among the substrates examined. It is notable that the enzyme was able to hydrolyze fibrous protein, such as gelatine, as well.

### 3.7. Kinetic

The *K*_*m*_ and *V*_max⁡_ values of the protease were determined using different concentrations of azocasein. The effect of increasing substrate concentration on the enzyme reaction rate follows a typical Michaelis-Menten equation with azocasein being the substrate. The *K*_*m*_ and *V*_max⁡_ values of the protease enzyme were calculated at 2.8 mg/mL and 31.20 U/mg of protein, respectively, at a pH of 8.0 and a temperature of 75°C (Figure 4(b)).

## 4. Conclusion

Our analyses suggest that the peel from red pitaya (*Hylocereus polyrhizus*) can be considered as an important candidate for the production of a protease enzyme with unique characteristics. It should be noted that the cost of the purified enzyme from pitaya peel (waste) was decreased around 74% compared with the commercial enzyme. The yield of the purified protease from the fruit is 71.3%. The enzyme is thermotolerant with enzymatic activity at high pH levels. In addition, the enzyme showed resilient stability in the presence of inhibitors, and surfactants and oxidizing agents. These features, as well as the abundant and cost effective source, indicate that this enzyme could be considered a useful protease enzyme in various industrial applications, such as the food and detergent industries.

## Figures and Tables

**Table 1:** Purification step of the thermoalkaline protease from *Hylocereus polyrhizus* peel.

Purification steps	Total protein (mg)	Total activity (U)	Specific activity (U/mg)	Purification fold	Yield (%)
Crude extract	44.2	557.2	12.6	1	100
Ammonium sulphate precipitation	3.9	462.4	118.4	9.4	83.2
Cation exchange chromatography	0.3	412.8	1312.9	104.2	74.1
Gel filtration chromatography	0.1	397.2	2787.1	221.2	71.3

Fold purification calculated with respect to the specific activity of the crude extract.

**Table 2:** Effect of metal ions, inhibitors, organic solvent, and surfactant and oxidizing agents on the protease activity.

Type	Agent	Concentration	Relative activity
	Noncomponents	—	100 ± 0.0^a^
Metal ions	Li^+^	10%	100 ± 0.1^a^
	K^+^	10%	100 ± 1.2^a^
	Na^+^	10%	100 ± 1.1^a^
	Sn^2+^	10%	100 ± 1.0^a^
	Ca^2+^	10%	125 ± 0.2^b^
	Mg^2+^	10%	110 ± 1.1^ab^
	Cu^2+^	10%	105 ± 0.5^ab^
	Fe^2+^	10%	52 ± 0.01^c^
	Zn^2+^	10%	38 ± 0.3^d^

Inhibitors	EDTA	10 mM	115 ± 0.3^ab^
	Ovomucoid	10 mM	100 ± 0.1^a^
	*β*-Mercaptoethanol	10 mM	100 ± 0.2^a^
	Iodoacetic acid	10 mM	100 ± 0.3^a^
	Bestatin	10 mM	100 ± 1.1^a^
	DTNB	10 mM	82 ± 0.0^ab^
	PMSF	10 mM	0.0 ± 1.1^e^

Organic solvent	Acetate	10%	100 ± 0.3^a^
	Ethanol	10%	100 ± 0.3^a^
	Isopropanol	10%	92 ± 0.2^d^
	Methanol	10%	83 ± 1.1^d^

Surfactant and oxidizing agents	Triton X-100	5%	100 ± 1.1^a^
	Tween-80	5%	100 ± 0.3^a^
	SDS	5%	73 ± 2.1^f^
	H_2_O_2_	2 M	62 ± 0.2^g^

The residual protease activity was determined after incubation of the enzyme with various phase components at room temperature for 1 h. The sample size for all experiments was three. Mean value followed by different letters differs significantly (*p* < 0.05).
